# Lower-body positive pressure diminishes surface blood flow reactivity during treadmill walking

**DOI:** 10.1186/s13104-019-4766-2

**Published:** 2019-11-08

**Authors:** Junichi Tajino, Akira Ito, Yusuke Torii, Koji Tsuchimoto, Hirotaka Iijima, Xiangkai Zhang, Momoko Tanima, Shoki Yamaguchi, Hiroshi Ieki, Ryosuke Kakinoki, Hiroshi Kuroki

**Affiliations:** 1 Department of Motor Function Analysis, Human Health Sciences, Graduate School of Medicine, Kyoto University, 53 Shogoin Kawahara-cho, Sakyou-ku, Kyoto City, Kyoto 606-8507 Japan; 2 Institute of Sport Science, ASICS Corporation, Kobe, Hyogo Japan; 3 Medical Division, Gunze Limited, Kyoto, Japan; 4 Department of Rehabilitation Medicine, Kyoto University Hospital, Kyoto University, Kyoto, Japan

**Keywords:** Gait, Cardiovascular, Safety, Design

## Abstract

**Objective:**

The purpose of this study was to determine the effects of the lower-body positive pressure on surface blood flow during standing still and treadmill walking to explore cardiovascular safety for application to rehabilitation treatment. Thirteen healthy volunteers participated in the experiment and surface blood flows were measured in the forehead, thigh, calf, and the top of the foot during standing still and walking under various pressure conditions (0 kPa, 5 kPa, and 6.7 kPa).

**Results:**

Lower-body positive pressure decreased the blood flow in the forehead and the thigh during walking (*p *< .05 for each), whereas an increasing trend in blood flow was observed during standing still (*p *< .05). Furthermore, in the forehead and thigh, the extent of blood flow increase at the onset of walking was found to decrease in accordance with the applied pressure (*p *< .01 for each). These findings suggest that during walking, lower-body positive pressure modulates the blood flow, which implies safeness of this novel apparatus for use during orthopedic rehabilitation treatment.

## Introduction

To optimize rehabilitation after injury in lower extremities (lower limbs), early re-ambulation is highly desirable, because a prolonged recovery might lead to gait problems [1–4]. However, in early re-ambulation, patients are vulnerable to falling or re-injury [5]. To counteract this, exercise devices or regimens are needed that can reduce loads on the injured body parts while keeping the appropriate motion.

Various walking aids [6], including suspension harness [7, 8] and swimming pools [1, 9] have been used to provide weight support. However, those regimens require gait optimizations [10, 11].

To modulate body-weight bearing [12], a treadmill device enclosed in an air chamber has been developed. By increasing the air pressure, known as the lower-body positive pressure, inside the chamber, it can provide body-weight support with minimal impacts on gait mechanics. This device is called lower-body positive pressure (LBPP) treadmill, which is being investigated for its various physiological effects during ambulation [13]. Heart rate during LBPP walking was found to be nearly similar to the resting heart rate [8]. Cutuk and coworkers showed that there were no significant changes in the range of motions in the knee and ankle during LBPP walking [14], which enables low-impact training without alterations in the movements [15]. Also, LBPP has little effects on the mean blood pressure [14, 16]. However, it remains unclear whether blood flow alteration occurs during LBPP walking, and there is limited evidence on this [17]. Given that fluctuation of blood flow could result in dizziness or ischemia in head and upper body, which might impair safety of gait practice [18], understanding the effects of LBPP on blood perfusion could be advantageous for patients undergoing orthopedic rehabilitation using LBPP treadmill.

The main purposes of this study was to examine the effects of LBPP on surface blood perfusion during walking and explore whether the blood perfusion is altered by different levels of exercises in LBPP.

## Main text

### Methods

#### Participants and experimental procedures

Thirteen healthy volunteers (male, 26.4 ± 5.6 years, 61.5 ± 7.03 kg) were recruited through the authors’ institution. We used a custom-made chamber treadmill to apply LBPP. As the pressure increased, the weight bearing of the participant inside the chamber decreased [19]. To secure air tightness, the participants wore a skirt-like object around their waists. The object was attached to the lid on the top of the chamber. After the participants’ weights in ambient pressure were measured as the baseline, extra pressure was applied. The participants walked at 1.1 m/s (4 km/h) speed in 1, 5, and 6.7 kPa conditions, following acclimatization periods of standing still in 1, 2, 3, 4, 5, 6, and 6.7 kPa for 1 min each. The 6.7 kPa is equivalent to 50 mmHg, which was considered as safe [14]. During the experiment, the participants’ weights were measured with load cells, and the percent weight bearing at each pressure (weight at each pressure/baseline) was calculated. Also, blood flow at four body points were recorded with a laser blood flowmeter (Cyber Farm, CDF-2000, Tokyo, Japan) at 500 Hz. The four body points were as follows: the forehead, one fingerbreadth above the center of the supraorbital ridge; the thigh, center of back side of the thigh; the calf, lower edge of the medial gastrocnemius muscle; and the top of the foot (instep), middle part of the third metatarsal bone. Moving averages at 10 Hz were obtained during each trial. For stability, the data during middle 30 s of each trial were incorporated in the analysis. In addition, the difference in blood flows between standing still and walking was calculated.

#### Statistical analyses

Obtained data were tested for homoscedasticity and normality using Levene’s test and Anderson–Darling test. To determine the changes depending on the pressure (0, 5, and 6.7 kPa) at four body points (forehead, thigh, calf, and instep), one-way factorial ANOVA was performed for standing still positions, walking, and assessing difference between standing still and walking. Furthermore, to compare the changes in blood flow between standing still and walking, two-way ANOVA was used, where standing/walking was the between-subject factor, and pressure level was the within-subject factor. Statistical significance was *p *< .05, with Tukey–Kramer adjustment for post hoc pairwise comparisons. JMP ver. 11 (SAS Institute, USA) was used for statistical analysis. Values in text and figures are presented as mean ± 95% confidence interval.

### Results

#### Decreased body weight with increasing pressure

An inverse relation was found between the applied pressure and the body weight. Percent weight bearing linearly decreased with increasing pressure during standing still (Additional file 1 : ANOVA, *p *< .001). Percent weight bearings at 5 and 6.7 kPa were 29.6 ± 8.2% and 12.0 ± 6.8%, respectively (Additional file 1: post hoc comparisons, *p *< .001 for 0 kPa vs. 5 kPa, and 0 kPa vs. 6.7 kPa, *p* = .02 for 5 kPa vs. 6.7 kPa).

#### Increased blood flow during standing still

**Fig. 1:**
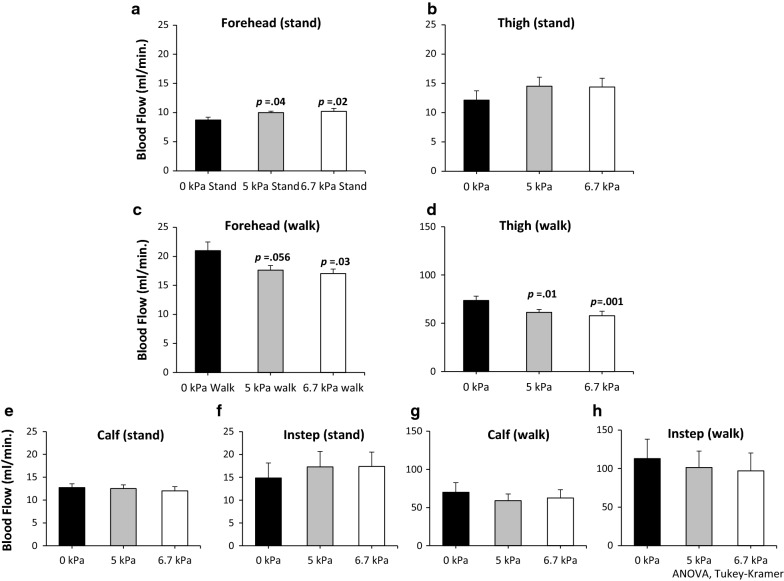
Blood flow during standing still and walking. During standing still, significant increase in blood flow is found in the forehead with increasing LBPP (**a** ANOVA, *p *= .011). Post hoc comparisons shows significant differences in 0 vs. 5 kPa and 0 vs. 6.7 kPa (**a** Tukey–Kramer, *p * = .04 and *p  *= .02 respectively). As for the thigh, difference in blood flow is not significant (**b** ANOVA, *p *= .256). During walking, significant decrease in blood flow is found in the forehead and the thigh with increasing LBPP (**c**, **d** ANOVA, *p *= .021, *p *= .001, respectively). In the forehead, trend and difference in blood flow are found in 0 vs. 5 kPa and 0 and 6.7 kPa (**c** Tukey–Kramer, *p *= .056, and *p *= .03, respectively). For the thigh, significant differences in blood flow are found in 0 vs. 5 kPa and 0 vs. 6.7 kPa (**d** Tukey–Kramer, *p *= .01, *p *= .001, respectively). As for the calf and the instep, differences in blood flows with increasing pressure are not significant both during standing still (**e**, **f** *p * > .05) and walking (**g**, **h** *p *> .3)

In the forehead, a significant increase in blood flow with increasing LBPP was observed (Fig. 1a: ANOVA, *p *= .01) during standing still. In addition, significant differences at 0 vs. 5 kPa (Fig. 1a: *p *= .04) and 0 vs. 6.7 kPa (*p *= .02) were found on pairwise comparisons.

For the thigh, the observed difference in blood flow with changing LBPP was not statistically significant (Fig. 1b: ANOVA, *p *= .256).

For the calf and instep, no significant differences in blood flows were found with changing LBPP (Fig. 1e: ANOVA, *p *> .05; and Fig. 1f: ANOVA, *p *> .05, respectively). Overall, it was found that blood flow during standing still increased in the forehead depending on the applied pressure.

#### Decreased blood flow during walking

During walking, blood flow in the forehead showed a significant decrease with increasing LBPP (Fig. 1c: ANOVA, *p* = .02). Post hoc tests revealed a significant difference between 0 and 6.7 kPa (Fig. 1c: *p *= .03). Although the *p* value was slightly higher than .5 (Fig. 1c: *p *= .056), there was a trend that blood flow at 5 kPa was lower than that at 0 kPa.

In the thigh, there was a significant decrease in blood flow with increasing LBPP (Fig. 1d: ANOVA, *p *= .001). Pairwise comparisons showed significant differences at 0 vs. 5 kPa and 0 vs. 6.7 kPa (Fig. 1d: *p *= .01 and *p *= .001, respectively). On the other hand, for the calf and instep, differences in blood flows were not significant (Fig. 1g, h: ANOVA, *p *> .05 for both). In short, the blood flow during walking decreased in the forehead and thigh depending on the extent of applied pressure.

#### Distinct slope of change between standing still and walking

**Fig. 2:**
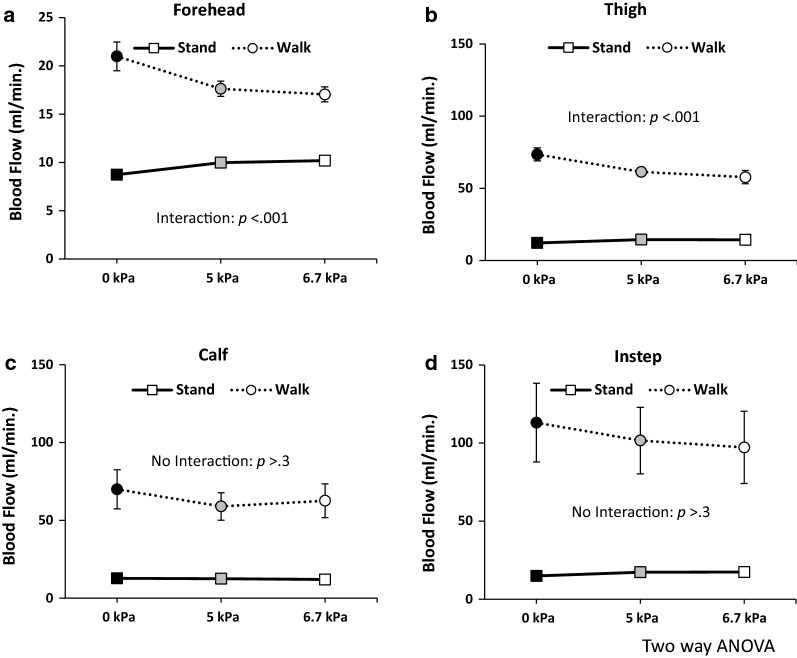
Comparisons of the slopes of the blood flow change. The forehead shows significant effect with changing postures (stand/walk). Also, significant interaction for posture × pressure is observed (**a** two-way ANOVA, *p *< .001 for both). For the thigh, significant effect with changing postures are observed. The thigh also shows significant interaction for posture × pressure (**b** two-way ANOVA, *p *< .001 for both). The calf and the instep show significant effects with changing postures (**c**, **d** two-way ANOVA, *p *< .001 for both); however, interactions for posture × pressure are not significant (**c**, **d** two-way ANOVA, *p *> .3 for both)

To demonstrate different slopes of blood flow change during standing still and walking in LBPP, two-way ANOVA was performed for each body part. In the forehead, there was a significant difference in blood flow between standing still and walking. A significant interaction between postures (standing/walking) and applied pressures was also observed (Fig. 2a: two-way ANOVA, *p *< .001 for both).

In the thigh, there was significant difference in blood flow between standing still and walking, and there was a significant interaction between postures and applied pressure (Fig. 2b: two-way ANOVA, *p *< .001 for both).

Concerning the calf and instep, although a significant difference in blood flow was found between standing still and walking (Fig. 2c, d: two-way ANOVA, *p *< .001 for both), no significant differences were observed between changing pressure or interactions between postures and pressure (Fig. 2c, d: two-way ANOVA, *p *> .3 for both).

#### Difference between standing and walking

**Fig. 3:**
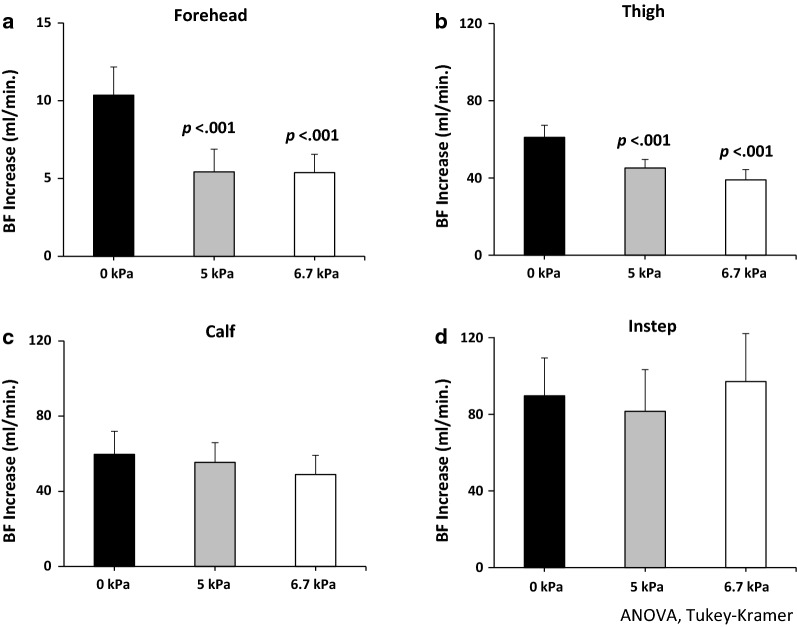
Extent of blood flow increase at the onset of the walking. In the forehead, the blood flow under 5 kPa and 6.7 kPa are significantly different from 0 kPa (**a** *p *< .001 for both). Also, in the thigh, significant differences in blood flow are found for 5 kPa and 6.7 kPa than for 0 kPa (**b** *p *< .001 for both). For the calf and the instep, no significant differences in blood flow are observed depending on applied pressure (**c**, **d** *p *> .05 for both) (ANOVA, Tukey–Kramer). *BF* blood flow; increase = Wwlking − standing

The extent of blood flow increase at the onset of walking was determined by using one-way ANOVA and post hoc comparisons. In the forehead, there were significant differences at 0 vs. 5 kPa and 0 vs. 6.7 kPa (Fig. 3a: *p *< .001 for both). Also, in the thigh, significant differences were found at 0 vs. 5 kPa and 0 vs. 6.7 kPa (Fig. 3b: *p* < .001 for both).

### Discussion

#### Overview

We investigated how LBPP affects surface blood perfusion during treadmill walking and standing still. Although musculoskeletal and kinematic influences of LBPP have been studied, it is still necessary to ensure no negative impact on cardiovascular system for its feasibility as a rehabilitation tool. In line with a previous study [14], our study showed that LBPP provides pressure-dependent weight support. We did not find acute disturbances in blood perfusion due to the LBPP.

#### Distinct change of blood flow during standing still and walking in LBPP

During walking, the blood flow in the forehead and thigh decreased with increasing pressure, whereas the blood flow increased during standing still by contrast. Although significant differences were not observed in other body parts, it is not surprising since blood flows at the end of lower extremities such as calf or foot fluctuate easily. Interestingly, blood flow in the forehead showed a different tendency between standing still and walking. Blood flow decreased during walking, whereas increased during standing still, which might be a consequence of the upward translocation of perfusion caused by LBPP [20]. Tanaka and colleagues regarding systematic cardiovascular response to LBPP reported that the R–R interval (interval between heart beats) increased under a 40 mmHg (5 kPa) pressure, indicating a slower heart rate [21]. Furthermore, Shi and coworkers studied effects of LBPP on baroreflex, which regulates heart rate and blood pressure on receiving signals from the baroreceptors located in aortic arch and carotid sinuses. They found that heart rate and carotid-vasomotor baroreflex were inversely related to the extent of LBPP, implying that the sensitivity of the baroreflex is restrained in accordance with LBPP [22]. This finding is consistent with those of other studies [22, 23]. Moreover, studies associated with space flight demonstrated that during and few days after habituation in space, which is analogous to LBPP environment, baroreflex sensitivity decreased compared with ground control rats [24] and humans [25]. Several possible factors might explain this inversed tendency. Nishiyasu and colleagues suggested that activation of mechanoreceptors by intramuscular pressure due to LBPP could enhance mean arterial blood pressure, via inhibition of baroreflex responsiveness [26]. Other studies also suggested baroreceptor inhibition through LBPP-induced intramuscular mechanoreceptors [27]. In addition, stimuli through muscle contraction during walking might reduce baroreceptor sensitivity combined with the effect of LBPP itself, as discussed above. Considering the low heart rate, combined effects of the attenuated baroreflex sensitivity due to the LBPP and exercise might explain the decrease in surface blood flows.

#### Increase of blood flow at the onset of walking

We also explored the extent of blood flow increase at the onset of walking to explain its escalation through physical activities under LBPP. We found a diminishing blood flow with LBPP in the forehead and thigh. This could be attributed to the opposite slopes of blood flow changes between standing still and walking (increasing and decreasing, respectively). Also, as mentioned in the previous studies [26, 27], intramuscular mechanoreceptors activated through LBPP might have attenuated the acute change in blood perfusion. Reduced blood perfusion might imply risk as less blood flow could induce dizziness [18]. However, considering the fact that cerebral artery velocity and cardiac output are less likely to be affected [28, 29] it could be suggested that LBPP has little or no adverse effects due to fluctuations in the cardiovascular response.

#### Advantages of LBPP

Despite the limitations of this study (discussed below), LBPP ambulation has several advantages over other methodologies, including no requirement for using upper body [14, 30], low impact [15], similarity in kinematic properties to ground exercises [9, 31], and gentleness to heart rate [8]. Besides, exercise duration in LBPP ambulation is short enough so that upward fluid shift and intravascular shear stress do not influence bone formation [32–34]. Considering the effects through mechanoreceptor [27, 35], these findings might imply that LBPP walking does not have catastrophic influences on blood perfusion, albeit requiring further affirmation.

In conclusion, we observed that walking in LBPP reduces blood flow elevation in the forehead and thigh during exercise. This finding suggests that LBPP provides weight support without catastrophic influences on cardiovascular parameters, through buffering acute increase of blood perfusion during exercise. Further studies would uncover potential applications of LBPP as well as implications of its blood flow modulation.

## Limitations

Since we only observed superficial blood flows in this study, we have three limitations as follows:

1. It is difficult to determine the effect of mechanostress in the lower body.
2. The magnitude of upward fluid translocation is unclear.
3. Influences of activated intramuscular mechanoreceptors on the autonomic system is still controversial [36].

## Supplementary information

**Additional file 1.** Percent weight bearing during standing still. The weight bearing is significantly decreased in accordance with the lower-body positive pressure (LBPP) (ANOVA, *p* <.001; Tukey-Kramer, *p* <.001 for 0 kPa vs. 5 kPa, and 0 kPa vs. 6.7 kPa; *p* =.02 for 5 kPa vs. 6.7 kPa).

## Data Availability

The datasets used and/or analyzed during the current study are not publicly available but are available from the corresponding author on reasonable request.

## Abbreviation

LBPP: lower body positive pressure
