# Supplementary material for: Lower-body positive pressure diminishes surface blood flow reactivity during treadmill walking
Source: BMC Res Notes. 2019 Nov 8;12:733. doi: 10.1186/s13104-019-4766-2 (PMC6839257; doi:10.1186/s13104-019-4766-2)
Supplement: Supplementary file 1 — Additional file 1. Percent weight bearing during standing still. The weight bearing is significantly decreased in accordance with the lower-body positive pressure (LBPP) (ANOVA, p <.001; Tukey-Kramer, p <.001 for 0 kPa vs. 5 kPa, and 0 kPa vs. 6.7 kPa; p =.02 for 5 kPa vs. 6.7 kPa). [file 13104_2019_4766_MOESM1_ESM.docx]

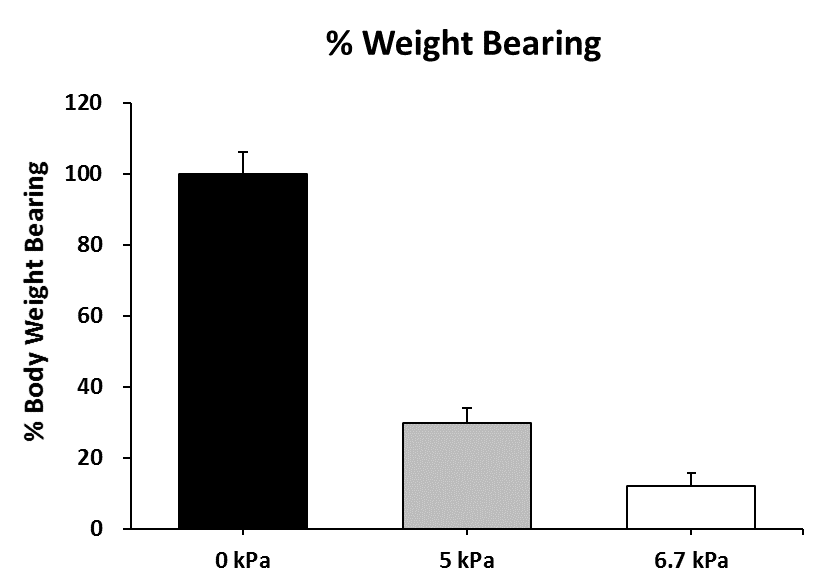


***p* <.001**

***p* <.001**

***p* =.02**

ANOVA, Tukey-Kramer

**Percent weight bearing during standing still:** The weight bearing was significantly decreased in accordance with the lower body positive pressure (LBPP) (ANOVA, *p* <.001; Tukey-Kramer, *p* <.001 for 0 kPa vs. 5 kPa, and 0 kPa vs. 6.7 kPa; *p* =0.02 for 5 kPa vs. 6.7 kPa).
